# Disruption of a primary health care domestic violence and abuse service in two London boroughs: interrupted time series evaluation

**DOI:** 10.1186/s12913-020-05397-x

**Published:** 2020-06-22

**Authors:** Jasmina Panovska-Griffiths, Alex Hardip Sohal, Peter Martin, Estela Barbosa Capelas, Medina Johnson, Annie Howell, Natalia V Lewis, Gene Feder, Chris Griffiths, Sandra Eldridge

**Affiliations:** 1 Department of Applied Health, Institute of Epidemiology and Health Care, University College London, London, UK; 2 Institute for Global Health, University College London, London, UK; 3 The Queen’s College, Oxford University, Oxford, UK; 4 Institute of Population Sciences, Queen Mary University London, London, UK; 5 IRISi, Bristol, UK; 6 Centre for Academic Primary Care, Bristol Medical School, University of Bristol, Bristol, UK

**Keywords:** Domestic violence and abuse, Interrupted time-series, Non-linear regression

## Abstract

**Background:**

Domestic violence and abuse (DVA) is experienced by about 1/3 of women globally and remains a major health concern worldwide. IRIS (*I*dentification and *R*eferral to *I*mprove *S*afety of women affected by DVA) is a complex, system-level, training and support programme, designed to improve the primary healthcare response to DVA. Following a successful trial in England, since 2011 IRIS has been implemented in eleven London boroughs. In two boroughs the service was disrupted temporarily. This study evaluates the impact of that service disruption.

**Methods:**

We used anonymised data on daily referrals received by DVA service providers from general practices in two IRIS implementation boroughs that had service disruption for a period of time (six and three months). In line with previous work we refer to these as boroughs B and C. The primary outcome was the number of daily referrals received by the DVA service provider across each borough over 48 months (March 2013–April 2017) in borough B and 42 months (October 2013–April 2017) in borough C. The data were analysed using interrupted-time series, non-linear regression with sensitivity analyses exploring different regression models. Incidence Rate Ratio (IRR), 95% confidence intervals and *p*-values associated with the disruption were reported for each borough.

**Results:**

A mixed-effects negative binomial regression was the best fit model to the data. In borough B, the disruption, lasted for about six months, reducing the referral rate significantly (*p* = 0.006) by about 70% (95%CI = (23,87%)). In borough C, the three-month service disruption, also significantly (*p* = 0.005), reduced the referral rate by about 49% (95% CI = (18,68%)).

**Conclusions:**

Disrupting the IRIS service substantially reduced the rate of referrals to DVA service providers. Our findings are evidence in favour of continuous funding and staffing of IRIS as a system level programme.

## Background

Domestic violence and abuse (DVA) includes threatening behaviour, violence or psychological, physical, sexual, financial, or emotional abuse between adults that are relatives, partners or ex-partners [1]. DVA is a violation of human rights with long term damage to health, experienced by one third of women globally [2]. In England and Wales an estimated 2 million women aged 16 to 59 years have experienced DVA in the year ending March 2018 with on average two women killed by their partner or ex-partner every week [3]. In addition to physical effects, women affected by DVA can also suffer chronic health problems including gynaecological problems, gastrointestinal disorders, neurological symptoms, chronic pain, cardiovascular conditions and mental health problems [4–7].

Over recent years, a consensus has been emerging that in order to improve the healthcare of women affected by DVA, greater health services involvement and better experiences of health services for these women is necessary [3, 8, 9]. For example, for improved delivery of sexual and reproductive health care, identification and management of DVA needs to be central not a tangential add-on service [10]. Therefore, commissioning of health services that are able to respond to DVA, should occur in a unified, coordinated fashion - not be fragmented between multiple organisations [11].

IRIS (*I*dentification and *R*eferral to *I*mprove *S*afety of women affected by DVA) is a, system-level, training and support programme, designed to improve the primary healthcare response to DVA [12]. The programme focuses on primary care clinicians identifying women who experience domestic abuse, discussing and offering, and, if the woman agrees to it, a referral to a named specialist within a DVA advocacy service.

A one-year cluster randomised controlled trial (RCT) evaluated the IRIS intervention in 24 intervention practices, with 24 control practices, across two areas (London Borough of Hackney and Bristol). IRIS substantially increased (2110%; 95%confidence interval CI = (1150,4240%)) the number of referrals to DVA service providers [12]. Trial data modelling showed that IRIS was cost-effective, with NHS and societal cost savings of £1 and £37 respectively per female patient aged 16 and over, per practice, per year [13]. IRIS has been found to be an acceptable intervention for both clinicians and patients [14, 15].

Implementation of IRIS outside of a trial [16] resulted in a large increase in referrals received by DVA service providers (3024%; 95%CI = (2055,4477%)), with no increase in 61 general practices in the fifth borough that did not fund IRIS, but instead provided DVA information sessions to which general practice clinicians were invited [17]. IRIS outside the trial setting is also cost-effective, from the NHS and societal perspective, good value for the NHS, cost saving for society – incremental net monetary benefit was £22 and £42 respectively [18]. A mixed method implementation process evaluation and a qualitative study found that staff mix and IRIS’ joined up approach, bridging the planets of general practice and specialist domestic abuse support services is crucial to making IRIS work [19, 20].

IRIS became a commissionable programme in 2010 and has been implemented across more than 40 different sites in England and Wales, with over 850 general practices fully IRIS trained to date that have referred over 14,000 women to specialist support through their GPs, with an estimated 50,000 women who have discussed DVA with a primary care clinician [21]. One quarter of areas that have commissioned IRIS since 2010 are no longer funding IRIS. Different local IRIS services have different referral rates with our process evaluation suggesting that short term funding and temporary IRIS service disruptions due to loss of trust in the service, results in drop in referral rates [19].

In our interrupted time series study [17], in two boroughs the provision of IRIS service was disrupted for a period of three and six months respectively. Our research question is whether the service disruption in each borough had an effect on the referral rate, during the period within which the disruption occurred. Using statistical analysis we thus quantify the impact of this disruption of IRIS as a service providing support to women affected by DVA, and hence learn lessons for future implementation of DVA programmes in health service contexts. The aim of this analysis is to determine whether transient IRIS service disruptions would decrease IRIS effectiveness, decreasing the referral rate of women affected by DVA, by clinicians to DVA workers.

## Methods

This study is a multidisciplinary collaboration of academic GPs, DVA specialists, qualitative and quantitative researchers.

### IRIS service description

IRIS core components include: 1. Practice based training to help identify women affected by DVA - two initial two-hour clinical sessions, with third for refresher training 2. Local GP delivering clinically relevant DVA training 3. IRIS advocate educator (AE) who receives referrals directly from trained clinicians, sees patients affected by DVA, usually within the practice, dispensing expert advocacy and ensuring direct access for women to specialist abuse services. Women can also self-refer if they see IRIS publicity material displayed within a practice [17].

### Data

For each borough, we included data from female patients aged 16 and above, registered at each general practice within the two boroughs. We used anonymised data on daily referrals received by DVA specialists from general practices in two boroughs, referred to as borough B and borough C in line with our previous work [17].

In borough B service disruption was due to funding of IRIS temporarily stopping, while in borough C funding was still in place but service disruption was due to the IRIS AE leaving with no replacement. In borough B, clinicians were told to refer women affected by DVA, to a different DVA service provider based in borough B. In borough C, clinicians were told to continue referring women affected by DVA to the local IRIS service, though these referrals were redirected to the host DVA service provider based in borough *C. iris* service provision was disrupted for a period of six and three months respectively in boroughs B and C. The dates of IRIS service disruption, implementation and data collection were collected for each borough (Table 1 and Table S2 in the Additional file 1).

**Table 1:** Timeline of IRIS data collection and mean referral rate across boroughs B and C over different time periods. We highlight the times of the data collection start and end, as well as the start and end of the service disruption in each borough. These times are labelled in Fig. 1(a)-(b)

Borough	Start date for data collection (***T***_**0**_)	Start date of IRIS implementation (***T***_**1**_)	Start date of IRIS service of disruption (***T***_**2**_)	End date of IRIS service disruption (***T***_**3**_)	End of IRIS data (***T***_**4**_)	Referral rate: mean [bias-corrected bootstrapped CI]			
						Over IRIS implementation period for which we have data (*T*_4_ − *T*_1_)	During period of IRIS implementation before disruption (*T*_2_ − *T*_1_)	During period of IRIS service disruption (*T*_3_ − *T*_2_)	Over period post IRIS service disruption (*T*_4_ − *T*_3_)
B	14.03.13 (t = 0)	14.03.14 (t = 365)	29.07.16 (t = 1234)	08.02.17 (t = 1428)	31.03.17 (t = 1479)	0.0344 [0.01965,0.0492]	0.04336 [0.0278,0.0589]	0.0023 [0.000551,0.00405]	0.005 [0.00032,0.0097]
C	02.10.13 (t = 0)	02.10.14 (t = 365)	05.08.16 (t = 1039)	31.10.16 (t = 1125)	25.03.17 (t = 1271)	0.0307 [0.0271,0.034]	0.0335 [0.0290,0.0379]	0.0156 [0.0073,0.0239]	0.0265 [0.0171,0.0362]

**Fig. 1:**
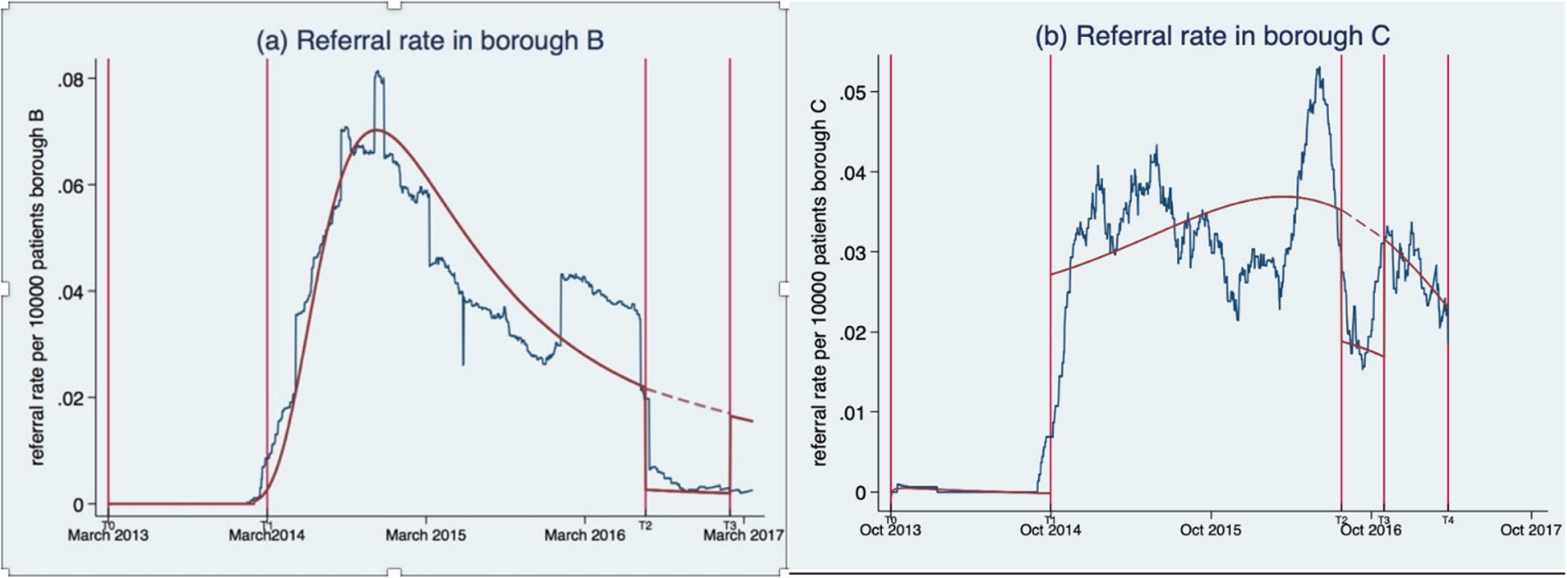
(**a**)-(**b**): Smoothened time series of the data from 73 GPs across two different boroughs (blue lines) and best fit fractional polynomial to the data (maroon solid and dashed lines) with equation and specific parameters outlined in the supplementary material. The graphs show the daily referral rate ($\frac{number\ of\ referrals}{GP\  size}\ast \mathrm{10,000}\Big)$) over the period for which we have data in borough B (in (**a**)) and borough C (in (b)). Boroughs B and C had a disruption of IRIS service for respectively six and three months (time period (*T*_3_ − *T*_2_) in (**a)**-(**b**)). The dashed lines in (**a**) and (**b**) illustrate the temporal trajectory of the fitted polynomials in the scenario where “no disruption of IRIS service” would have occurred in these boroughs **b** and **c**

The primary outcome was the number of daily referrals received by the DVA service provider from each of the 36 and 37 general practices in boroughs B and C respectively over 48 months (March 2013 and April 2017) in borough B and 42 months (October 2013–April 2017) in borough C (details in Table 1). Table 1 shows for each borough, the start date of data collection, the start of IRIS implementation, the start of the IRIS service disruption, the end of IRIS service disruption and the end date of data collection (respectively times *T*_0_, *T*_1_, *T*_2_, *T*_3_ and *T*_4_), with the average referral rate in the periods before, during and after IRIS service interruption. *T*_0_, *T*_1_, *T*_2_, *T*_3_ and *T*_4_ are also labelled in Fig. 1 (a)-(b).

### Statistical analysis

The outcome of interest was the number of daily referrals received by the DVA service provider from general practices, with the rate per 10,000 patients calculated as $\frac{number\ of\ referrals}{practice\ size}\ast \mathrm{10,000}$. We modelled this outcome separately for each borough and testing different regression models (negative binomial, mixed-effect negative binomial models or mixed-effect Poisson model- details in Additional file 1). Practice size was included in the model as an offset term. The model allowed for differences in referral rates between GP practices via a random intercept for GP practice. Since the daily number of referrals contained a large proportion of zeroes, we also assessed whether a zero-inflated mixed effects negative binomial model or a zero-inflated mixed effects Poisson model improved the fit to the data. For each regression model, we calculated the Akaike Information Criterion (AIC) and the Bayesian Information Criterion (BIC) to compare models. The best-fit model was chosen based on the smallest values of these quantities (details are in Table S1 of Additional file 1).

Exploratory analysis showed that the referral rate during the IRIS implementation period was not constant over time, even outside the period of interruption. We therefore modelled the post-implementation trend of the referral rate as a non-linear function of time. This allows us to derive a model-based estimate of the referral rate over the whole period under consideration in this analysis. By adding an indicator variable for days falling into the disruption period, we could estimate the difference in the referral rate due to the interruption during the period within which it occurred. Our model assumes that the effect of the disruption on the referral rate is immediate, suggesting that even transient IRIS service disruptions would immediately raise the barrier for GPs to make referrals for DVA.

We used fractional polynomials, with two time transformations as well as an indicator variable for the disruption period, to identify the optimal transformations of time for our models separately for each borough (Model 1 in Additional file 1 with details of the transformations in Tables S3 and S4). For graphical display, we smoothed the observed average daily referral rate over all practices using a moving average with uniform weights (101 and 45 lagged and forward terms for each referral respectively in boroughs B and C respectively).

### Sensitivity analyses

To investigate the robustness of our model fit and account for different ways of modelling temporal fluctuations in the referral rate, we conducted a sensitivity analysis for each borough by fitting both a simpler and a more complex model in comparison to Model 1. The simple model (Model 2 in Additional file 1) assumes that the referral rate is constant over time, other than during the disruption period, and calculates and tests the simple difference in the average referral rate between the implementation and disruption periods, albeit controlling for between-practice differences in the base referral rate. Within this Model 2, the model included two predictors: one time transformation, as the random intercept for time, and the indicator variable for the disruption period (see Additional file 1 for details). In contrast, for the more complex model (Model 3 in the Additional file 1), we allowed 5 predictors within the mixed effects negative binomial model: four time transformations as well as the indicator variable for the disruption period. By allowing the fractional polynomials to have higher number of terms, we allowed a closer fit of the modelled referral rate to the observed referral rate over time (see Additional file 1 for details).

For each of the Models 1–3, and for both boroughs B and C, we calculated the incidence rate ratio (IRR), their 95% CI and the *p*-value, quantifying the impact and significance of the IRIS service disruption (details in Tables S3 and S4 in Additional file 1). To add robustness to the results, we added bootstrapped calculations for the standard errors with 500 replications. All analyses were done in STATA version 15.1.

## Results

### Descriptive results

Table 1 shows the mean referral rates over all practices in each borough in the periods before, during, and after disruption of the IRIS service illustrating that the mean referral rate is reduced during the disruption period in both boroughs. The mixed-effect negative binomial model was the best-fit model for the data in both boroughs, since both AIC and BIC were minimal for this model (see Table S1 in Additional file 1 for details). The best-fit models superimposed over the corresponding smoothed time-series of the data for boroughs B and C are shown in Fig. 1(a)-(b). Descriptively, in both boroughs we see a steep increase of the referral rate after the start of the IRIS intervention. The referral rate then remains high for a few months, before declining over time in both boroughs. For a few months before the disruption period, there is a surge in referral rates in both boroughs, before a sharp decline in both boroughs during the disruption period. The referral rate stays approximately stable during the disruption period with referral rate recovering to pre-suspension levels in borough C, but not in borough B, where it remains low.

### Estimated effect of the service disruption

The estimated IRR for the effect of the suspension in each borough are shown in Table 2. In borough B, our model estimated an IRR of 0.301 (95% CI = (0.128, 0.774), *p* = 0.006). Thus we estimate that the referral rate was reduced by about 70% (between 23 and 87%) during the disruption period, compared to what it would have been without the service disruption.

**Table 2:** Results from the statistical analysis showing the impact of the interruption of IRIS service (IRR) and the p-value of the IRIS service interruption

Borough	Observed coefficient	Bootstrap Standard error	IRR [95% CI]	*p*-value
B	−1.202	0.434	0.301 [0.128,0.774]	0.006
C	−0.667	0.237	0.513 [0.322,0.817]	0.005

In borough C, our model estimated an IRR of 0.513 (95% CI = (0.322,0.817), *p* = 0.005). Thus we estimate that the referral rate was reduced by about 49% (between 18 and 68%) during the disruption period, compared to what it would have been without the service disruption.

### Sensitivity analyses

For both boroughs, our chosen model fitted the data best, the simpler model being underfitted, and the complex model being overfitted (Tables S3 and S4 in Additional file 1). However, in each borough all three models gave approximately equivalent results: both in terms of the IRRs and their 95% CI (see Table S3 and S4 in Additional file 1) and the significant difference in the referral rate during the disruption period (*p*-value is less than 0.05 across all three models – see Tables S3-S4 for details).

## Discussion

Our results are evidence that temporary disruption of IRIS, as a programme providing support to women affected by DVA, had a substantial effect on referrals of women affected by DVA to specialist services in both implementation boroughs. In borough B, disruption lasted for six months. The referral rate to DVA specialist services was reduced by 70% during the disruption and did not recover after the disruption. In borough C, the disruption was shorter, lasting three months. During this disruption, the referral rate was reduced by 49%. However, in this borough, although the disruption reduced the referral rate, this reduction was temporary. Once the disruption stopped, the mean referral rate recovered to almost pre-disruption levels (Table 1 and Fig. 1(b)).

Since these disruptions were substantial regardless of their length, our study provides evidence that sustainability of a DVA programme in general practice requires on-going reinforcement strategies and processes in place, not just from the outset but also continually as the programme progresses. For the IRIS programme, this requires the physical presence in general practices of the IRIS AE – without which, as seen in borough C, even short IRIS service disruption that clinicians were unaware of substantially reduced referrals. This is unsurprising, as DVA remains largely an invisible issue in clinical consultations and society.

The challenge in the current health care commissioning and financial climate in the United Kingdom is to ensure that DVA is made visible, with IRIS programmes prioritised in local health policy and wider needs assessments. IRIS offers a cost effective and evidence-based solution along with simple, specialised and effective referral pathways. It should be funded and sustained as routinely as other health care services.

This is the first study that quantifies the impact and significance of disrupting a system-level programme that offers support to woman who experience DVA outside of a trial setting. The analysis we report here extends our previous multi-disciplinary research utilising a rich data set of DVA referrals from a large number of practices across multiple London boroughs [17, 19]. We have applied ITS and non-linear regression analysis to make predictions from a data set comprising of DVA referrals from a large number of practices across two London boroughs. This is accepted as a robust and efficient method for evaluation of public health and primary care evaluations [22, 23].

Whilst regression modelling is useful in drawing conclusion for the duration of the study where fitted curves mimic the data, the presence of turning points in the non-linear fits makes them unreliable for prediction beyond the period for which data are available. An alternative would be to develop and utilise dynamic temporal models that use the data to calibrate the equations to the historic pattern, and then be used to make future prediction.

As further data on the DVA referrals in IRIS implementation settings become available, further analysis can explore the longer-term impact of the intervention. Comparing IRIS implementation across different settings would be an interesting extension of this work. Furthermore, evaluating adapted versions of the IRIS model that are currently being piloted is a feasible extension of this work. For example, following a successful pilot in two sexual health clinics, further work is ongoing to develop this into a commissionable service [24, 25].

## Conclusions

Disrupting the IRIS primary healthcare domestic violence programme substantially reduced the rate of referrals to DVA service providers. Our work yields important lessons for the implementation of health care-based DVA programmes in general and specifically for future IRIS implementation in the UK, revealing the negative and enduring impact of disrupting a primary care service for woman who experience DVA, highlighting the need for continual support and funding of such service provision.

## Supplementary information

**Additional file 1:** Supplementary material for paper entitled: Disruption of a primary health care domestic violence and abuse service in two London boroughs: interruptedtime series evaluation. Appendix A: Details of the statistical and sensitivity analysis.

## Data Availability

The datasets used and analysed during this study and the numerical codes used to generate the outcomes of this paper are available from the corresponding author on reasonable request.

## Abbreviations

DVA: Domestic violence and abuse
IRIS: Identification and Referral to Improve Safety
GP: General practitioner
NHS: National Health Service
IRR: Incidence rate ratio
ITS: Interrupted time series
RCT: Randomised control trial
AIC: Akaike Information Criterion
BIC: Bayesian Information Criterion
CI: Confidence interval
AE: Advocate educator
