# Supplementary material for: Disruption of a primary health care domestic violence and abuse service in two London boroughs: interrupted time series evaluation
Source: BMC Health Serv Res. 2020 Jun 22;20:569. doi: 10.1186/s12913-020-05397-x (PMC7309975; doi:10.1186/s12913-020-05397-x)
Supplement: Supplementary file 1 — Additional file 1: Supplementary material for paper entitled: Disruption of a primary health care domestic violence and abuse service in two London boroughs: interruptedtime series evaluation. Appendix A: Details of the statistical and sensitivity analysis. [file 12913_2020_5397_MOESM1_ESM.docx]

**Supplementary material for paper entitled:** Disruption of a primary health care domestic violence and abuse service in two London boroughs: interrupted time series evaluation

**Appendix A: Details of the statistical and sensitivity analysis**

**Overview**

This appendix gives the details of the statistical analysis on the anonymised data from daily referrals received by DVA service providers from 36 GPs in boroughs B and 37 GPs in borough C between the period of October 2014 and April 2017.

**Methods**

The data was coded using STATA software (version 15.1). For each borough analysis comprised the following steps:

1. Time series was constructed from the number of daily referrals across all GPs in the borough. We used fractional polynomials to derive time transformation for the best fit negative binomial model to the data, also including a predictor variable for the disruption of IRIS service over some period.

2. Using the same time transformations, and a disruption predictor variable, we compared a negative binomial model, a zero-inflated negative binomial model, a the zero-inflated Poisson model and a mixed-effects negative binomial models projecting the corresponding Akaike Information Criterion (AIC) and Bayesian Information Criterion (BIC) values. Based on these values we chose the best-fit model for each borough.

3. For the best-fit model in each borough, we projected outcome variables: incidence rate ratios (IRRs) with 95% CI and the p-value for the disruption predictor variable.

4. We explored, in each borough, whether the outcome variables are affected by the model fit. To do this we increased and decrease the number of time transformation used, accounting for more or less temporal oscillations in the fitted model.

5. For the most appropriate model, we then checked robustness of the results by generating bootstrapped outcome values with 500 replications.

**Results**

1. In each borough, using fractional polynomials we identified the best-fit model to be

$log\left( \hat{Y}_{ij} \right)=\left( \beta_{0}+ u_{0j} \right)+ \beta_{1}t_{1}\left( {time}_{ij} \right)+\beta_{2}t_{2}\left( {time}_{ij} \right)+\gamma{DISR}_{ij}+log\left( \frac{{PracticeSize}_{ij}}{10000} \right) , u_{0j}\sim N(0, \sigma_{u}^{2})$ (1)

where $\hat{Y}_{ij}$ is the estimated number of referrals for practice *j* at time *i*, assumed to follow a negative binomial distribution, $u_{0j}$ is a random intercept term, which varies between practices, $t_{1}$ and $t_{2}$ are transformations of time identified via fractional polynomials with $t_{1}\left( time_{ij} \right)=\left( \frac{time_{ij}}{1000} \right)^{-2}-1.17426, t_{2}\left( time_{ij} \right)=\left( \frac{time_{ij}}{1000} \right)^{-2}\log\left( \frac{time}{1000} \right)+0.0943$ in borough B and $t_{1}\left( time_{ij} \right)=\left( \frac{time_{ij}}{100} \right)^{3}-548.9608, t_{2}\left( time_{ij} \right)=\left( \frac{time_{ij}}{100} \right)^{3}\log\left( \frac{time}{100} \right)-1154.2865$ in borough C, ${DISR}_{ij}$ is an indicator variable coded 0 for days at which IRIS was running, and 1 for days during the disruption period.

2. Comparing different regression models

The calculated AIC and BIC values, using the same time transformations as in (1), for a negative binomial model, a zero-inflated mixed effects negative binomial model, a zero-inflated mixed effects Poisson model and a mixed-effects negative binomial model are presented in Table S1. Based on the smallest AIC and BIC values, for both boroughs B and C, the mixed-effects negative binomial regression model was the most appropriate model.We refer to this as Model 1.

| Borough | Negative binomial model | | Zero-inflated negative binomial model | | Zero-inflated Poisson model | | Mixed-effects negative binomial model | |
| --- | --- | --- | --- | --- | --- | --- | --- | --- |
|  | AIC | BIC | AIC | BIC | AIC | BIC | AIC | BIC |
| B | 2953.771 | 2996.901 | 2980.063 | 3040.444 | Does not converge | | 2883.169 | 2883.93 |
| C | 4064.116 | 4106.074 | 4083.481 | 4097.223 | 4073.001 | 4120.134 | 3843.929 | 3894.286 |

Table S1: Results from comparing different regression models that fit the data in boroughs B and C.

3. Using the mixed-effects negative binomial model (i.e. Model 1) in each borough, with predictor variables for the time transformations and the disruption of IRIS service, the IRRs and their 95% CI and the corresponding p-values for the disruption predictor variables are given in Table S2. Results suggest that the disruption was both impactful and significant across boroughs B and C.

| Borough | Mixed-effects negative binomial model with two time transformations (Model 1) | | |
| --- | --- | --- | --- |
|  | IRR | 95% CI | p-value |
| B | 0.3013 | (0.1279, 0.7741) | 0.006 |
| C | 0.5138 | (0.3218, 0.8168) | 0.005 |

Table S2: Projections from the mixed-effects negative binomial model from equation (1) for each borough.

4. We explored two variations of Model 1:

Model 2 = Simpler model: We fitted a mixed-effects negative binomial regression, including predictors for the random intercept for time (i.e. only one time transformation) and the indicator variable for the disruption period. Model 2 equation is therefore

$log\left( \hat{Y}_{ij} \right)=\left( \beta_{0}+ u_{0j} \right)+ \beta_{1}time_{ij}+\gamma{DISR}_{ij}+log\left( \frac{{PracticeSize}_{ij}}{10000} \right) , u_{0j}\sim N(0, \sigma_{u}^{2})$ (2)

Model 3 = More complex model: We fitted a more complex mixed-effects negative binomial regression, including up to four time transformations as well as the indicator variable for the suspension period as predictors. The equation for Model 3 is therefore

$log\left( \hat{Y}_{ij} \right)=\left( \beta_{0}+ u_{0j} \right)+ \beta_{1}t_{1}\left( {time}_{ij} \right)+\beta_{2}t_{2}\left( {time}_{ij} \right)+\beta_{4}t_{4}\left( {time}_{ij} \right)+\beta_{5}t_{5}\left( {time}_{ij} \right)+\gamma{DISR}_{ij}+log\left( \frac{{PracticeSize}_{ij}}{10000} \right) , u_{0j}\sim N(0, \sigma_{u}^{2})$ (3)

where $t_{1},t_{2},t_{3}$ and $t_{4}$ are transformations of time identified via fractional polynomials. In borough B these time transformations are $t_{1}\left( time_{ij} \right)=\left( \frac{time_{ij}}{1000} \right)^{-2}-1.17426, t_{2}\left( time_{ij} \right)=\left( \frac{time_{ij}}{1000} \right)^{-2}\log\left( \frac{time}{1000} \right)+0.0943, t_{3}\left( time_{ij} \right)=\left( \frac{time_{ij}}{1000} \right)^{0.5}, t_{4}\left( time_{ij} \right)=\left( \frac{time_{ij}}{1000} \right)^{0.5}\log\left( \frac{time}{1000} \right)$ and in borough C these are $t_{1}\left( time_{ij} \right)=\left( \frac{time_{ij}}{100} \right)^{3}-548.9608, t_{2}\left( time_{ij} \right)=\left( \frac{time_{ij}}{100} \right)^{3}\log\left( \frac{time}{1000} \right)-1154.2865$, $t_{3}\left( time_{ij} \right)=\left( \frac{time_{ij}}{1000} \right)^{-0.5}, t_{4}\left( time_{ij} \right)=\left( \frac{time_{ij}}{1000} \right)^{-4}$

The AIC and BIC values, the IRRs and the p-values for Models 1-3 are similar, as shown in Tables S3 and S4, and all three models suggest a statistically significant decline in referral rate during the disruption period, with a very small difference in the p-value. Therefore, we decided there was no added value in adding complexity to the fit with using Model 3 that evidently overfits the data. So, we decided to use Model 1 as the best-fit model for each borough.

5. We used Model 1 with bootstrap calculations and 500 replications to generate the results that are presented in Table 2 in the main manuscript.


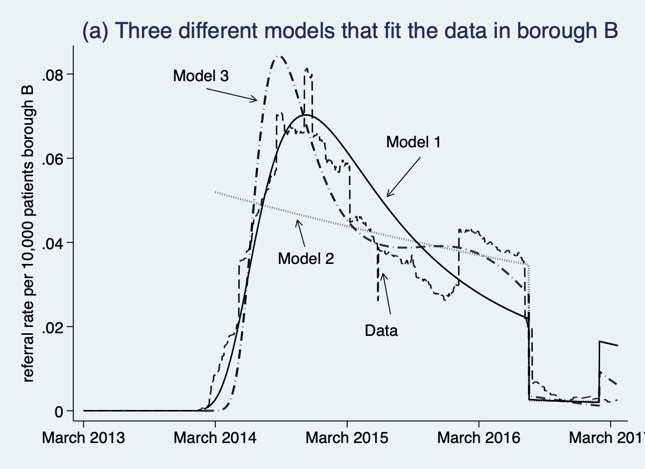

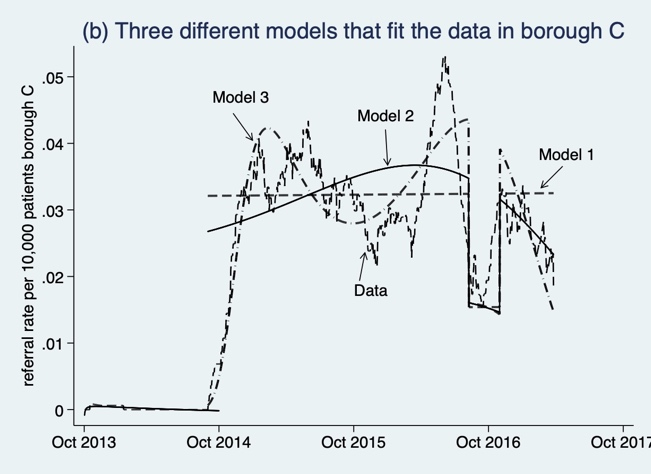


Figure S1: (a) Graphical illustration of the fitting to the data (dashed line) from borough B of Model 1 (black solid line), Model 2 (grey solid line) and Model 3 (dash-dot line) defined by equations (1)-(3) with parameters in Table S3. (b) Graphical illustration of the fitting to the data in borough C (dashed line) of Model 1 (black solid line), Model 2 (grey solid line) and Model 3 (dash-dot line) defined by equations (1)-(3) in borough B and C, with relevant parameters given in Tables S3 and S4.

| Model | coefficients | Standard error | AIC | BIC | IRR  (95% CI) | p-value |
| --- | --- | --- | --- | --- | --- | --- |
| Model 1 |  |  | 2830.252 | 2883.93 | 0.3013 (0.1279,0.7741) | 0.006 |
|  | $\beta_{0}$=-13.62 | 0.259 |  |  |  |  |
|  | $\beta_{1}$=2.206 | 0.313 |  |  |  |  |
|  | $\beta_{2}$=2.148 | 0.316 |  |  |  |  |
|  | $\gamma$=-1.199 | 0.411 |  |  |  |  |
| Model 2 | $\beta_{0}$=12.709 | 0.311 | 2870.783 | 2913.917 | 0.2079 (0.0938,0.4611) | 0.000 |
|  | $\beta_{1}$=-0.001 | 0.000235 |  |  |  |  |
|  | $\gamma$=-1.57 | 0.406 |  |  |  |  |
| Model 3 |  |  | 2832.169 | 2899.266 | 0.3222 (0.1312,0.7963) | 0.014 |
|  | $\beta_{0}$=-277.464 | 177.966 |  |  |  |  |
|  | $\beta_{1}$=22.447 | 8.895 |  |  |  |  |
|  | $\beta_{2}$=14.231 | 5.3245 |  |  |  |  |
|  | $\beta_{3}$=266.339 | 119.058 |  |  |  |  |
|  | $\beta_{4}$=-103.803 | 46.881 |  |  |  |  |
|  | $\gamma$=-1.132 | 0.462 |  |  |  |  |

Table S3: Comparing Models 1-3 and their projections in borough B.

| Model | coefficients | Standard error | AIC | BIC | IRR  (95% CI) | p-value |
| --- | --- | --- | --- | --- | --- | --- |
| Model 1 |  |  | 3828.436 | 3894.286 | 0.5131 (0.3218, 0.8168) | 0.005 |
|  | $\beta_{0}$=-13.122 | 0.257 |  |  |  |  |
|  | $\beta_{1}$=0.00389 | 0.00204 |  |  |  |  |
|  | $\beta_{2}$=-0.00156 | 0.0008 |  |  |  |  |
|  | $\gamma$=-0.667 | 0.243 |  |  |  |  |
| Model 2 |  |  | 3846.545 | 3888.508 | 0.5268 (0.3282,0.8457) | 0.008 |
|  | $\beta_{0}$=-13.153 | 0.299 |  |  |  |  |
|  | $\beta_{1}$=-0.001 | 0.002 |  |  |  |  |
|  | $\gamma$=-0.641 | 0.241 |  |  |  |  |
| Model 3 |  |  | 3843.929 | 3895.578 | 0.389 (0.2378,0.6392) | 0.000 |
|  | $\beta_{0}$=-41.297 | 6.622 |  |  |  |  |
|  | $\beta_{1}$=0.6159 | 0.014 |  |  |  |  |
|  | $\beta_{2}$=-0.0217 | 0.0049 |  |  |  |  |
|  | $\beta_{3}$=25.6403 | 6.0199 |  |  |  |  |
|  | $\beta_{4}$=-0.155 | 0.0379 |  |  |  |  |
|  | $\gamma$=-0.942 | 0.252 |  |  |  |  |

Table S4: Comparing Models 1-3 and their projections in borough C.

Additional post-hoc analysis:

In response to reviewer’s comments we have fitted the data from borough B to Model 4 that comprises two parts:

1) part 1 fitted on the time period time=[0,1019] days

2) part 2 fitted on the time period time=[1020,1479] days that includes the disruption period in the time=[1234, 1428] days.

These submodels are shown in Figure S2, split by the black vertical line.

For both sub-models part 1 and part 2 of Model 4 we fitted a mixed-effects negative binomial regression, including predictors for the random intercept for time with two time transformations $t_{1}\left( time_{ij} \right)=\left( \frac{time_{ij}}{1000} \right)^{-2}-1.17426, t_{2}\left( time_{ij} \right)=\left( \frac{time_{ij}}{1000} \right)^{-2}\log\left( \frac{time}{1000} \right)+0.0943$ and the indicator variable for the disruption period but over different time periods. This is the same as per all the other models (Models 1-3) for borough B. Hence we remain consistent in our analysis. Specifically, Model 4 part 1 equation is therefore

$log\left( \hat{Y}_{ij} \right)=\left( \beta_{0}+ u_{0j} \right)+ \beta_{1}time_{ij}+\gamma{DISR}_{ij}+log\left( \frac{{PracticeSize}_{ij}}{10000} \right) , u_{0j}\sim N(0, \sigma_{u}^{2})$ (4)

while Model 4 part 2 equation is

$log\left( \hat{Y}_{ij} \right)=\left( \beta_{0}+ u_{0j} \right)+ \beta_{1}time_{ij}+log\left( \frac{{PracticeSize}_{ij}}{10000} \right) , u_{0j}\sim N(0, \sigma_{u}^{2})$ (5)

with the parameters listed in table S4.

As evident from the table, the overall AIC and BIC for Model 4 is larger than those for Models 1-3 in Table S3. Hence Model 2 is a better overall fit to the data. The impact of the disruption projected by Models 1 and 4 is very similar, with Model 4 suggesting significant (p=0.007) decrease in referral of 72% (95%CI=(25%,88%)) with disruption, while Model 2 is suggesting significant (p=0.006) decline in referral of 70% (95%CI=(23%,87%)) with disruption. The difference in predictions is thus very marginal.

| Model | coefficients | Standard error | AIC | BIC | IRR  (95% CI) | p-value |
| --- | --- | --- | --- | --- | --- | --- |
| Model 4 (both parts) |  |  | 2856.071 | 2943.213 |  |  |
| Model 4  Part 1  time=[0,1019] |  |  |  |  |  |  |
|  | $\beta_{0}$=-13.596 | 0.269 | 2241.20 | 2281.89 |  |  |
|  | $\beta_{1}$=2.302 | 0.433 |  |  |  |  |
|  | $\beta_{2}$=2.247 | 0.413 |  |  |  |  |
|  |  |  |  |  |  |  |
| Model 4  Part 2  time=[1020,1079] |  |  | 614.871 | 661.323 | 0.278(0.1087,0.7102) | 0.007 |
|  | $\beta_{0}$=-15.874 | 1.551 |  |  |  |  |
|  | $\beta_{1}$=13.163 | 7.052 |  |  |  |  |
|  | $\beta_{2}$=37.643 | 21.829 |  |  |  |  |
|  | $\gamma$=-1.2807 | 0.478 |  |  |  |  |

Table S4: Comparing Model 4, that fits two sub-models parts 1 and part 2 to the data and their projections in borough B. This additional analysis was undertaken at request of one of the reviewers.


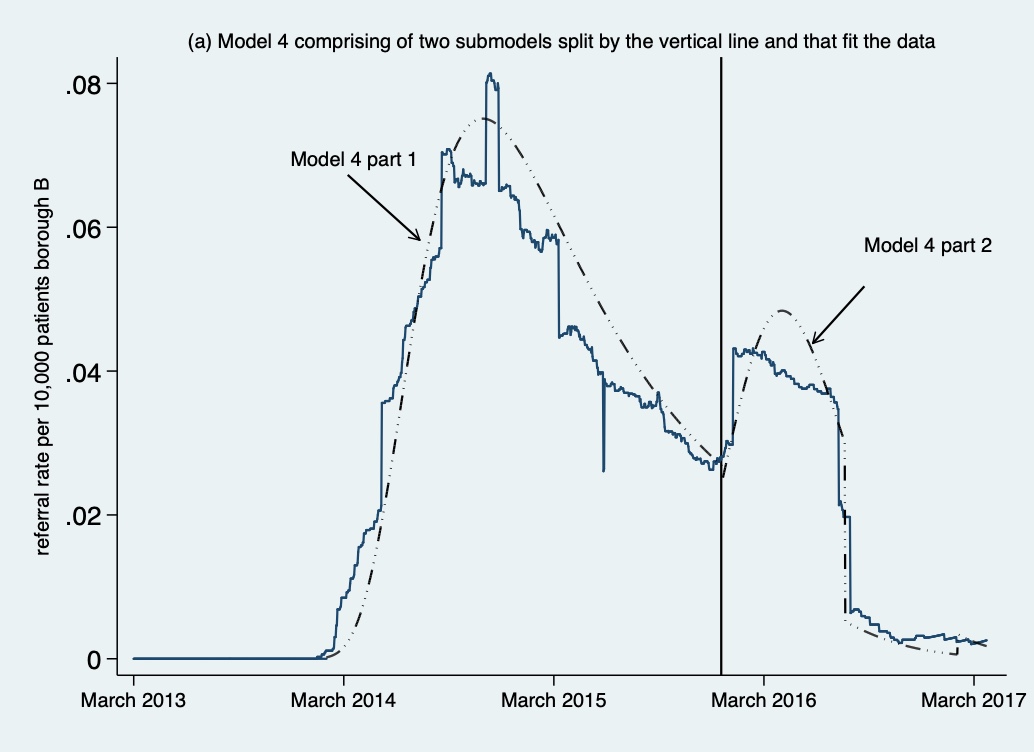


Figure S2: Graphical illustration of the fits to the data Model 4 parts 1 and 2, defined by equations (4)-(5) in borough B.
